# The Case for Adopting the “Species Complex” Nomenclature for the Etiologic Agents of Cryptococcosis

**DOI:** 10.1128/mSphere.00357-16

**Published:** 2017-01-11

**Authors:** Kyung J. Kwon-Chung, John E. Bennett, Brian L. Wickes, Wieland Meyer, Christina A. Cuomo, Kurt R. Wollenburg, Tihana A. Bicanic, Elizabeth Castañeda, Yun C. Chang, Jianghan Chen, Massimo Cogliati, Françoise Dromer, David Ellis, Scott G. Filler, Matthew C. Fisher, Thomas S. Harrison, Steven M. Holland, Shigeru Kohno, James W. Kronstad, Marcia Lazera, Stuart M. Levitz, Michail S. Lionakis, Robin C. May, Popchai Ngamskulrongroj, Peter G. Pappas, John R. Perfect, Volker Rickerts, Tania C. Sorrell, Thomas J. Walsh, Peter R. Williamson, Jianping Xu, Adrian M. Zelazny, Arturo Casadevall

**Affiliations:** a Laboratory of Clinical Infectious Diseases, NIAID, NIH, Bethesda, Maryland, USA; b University of Texas Health Science Center at San Antonio, San Antonio, Texas, USA; c Molecular Mycology Research Laboratory, University of Sydney, Sydney, Australia; d Westmead Institute for Medical Research, Westmead, New South Wales, Australia; e Broad Institute of MIT and Harvard, Cambridge, Massachusetts, USA; f Office of Cyber Infrastructure and Computational Biology, NIAID, NIH, Bethesda, Maryland, USA; g Institute of Infection and Immunity, St. George’s University of London, London, United Kingdom; h Colombia Instituto Nacional de Salud, Bogota, Colombia; i Mycology Center, Changzheng Hospital, Second Military Medical University, Shanghai, China; j Dipartimento di Scienze Biomediche per la Salute, Università degli Studi di Milano, Milan, Italy; k Institut Pasteur, Molecular Mycology Unit, Paris, France; l School of Biological Sciences, University of Adelaide, Adelaide, Australia; m Los Angeles Biomedical Research Institute, Harbor-UCLA Medical Center, Los Angeles, California, USA; n Department of Infectious Disease Epidemiology, Imperial College London, London, United Kingdom; o Nagasaki University, Nagasaki, Japan; p Michael Smith Laboratories, University of British Columbia, Vancouver, Canada; q Instituto Nacional de Infectologia Evandro Chagas, Fiocruz, Rio de Janeiro, Brazil; r Department of Medicine, University of Massachusetts Medical School, Worcester, Massachusetts, USA; s Institute of Microbiology and Infection and School of Biosciences, University of Birmingham, Birmingham, United Kingdom; t Faculty of Medicine, Siriraj Hospital, Mahidol University, Bangkok, Thailand; u Division of Infectious Diseases, University of Alabama at Birmingham, Birmingham, Alabama, USA; v Duke University School of Medicine, Durham, North Carolina, USA; w Robert Koch Institut, Berlin, Germany; x Marie Bashir Institute for Infectious Diseases and Biosecurity, University of Sydney, Sydney, Australia; y Departments of Medicine, Pediatrics, and Microbiology and Immunology, Weill Cornell Medicine, New York, New York, USA; z Department of Biology, McMaster University, Hamilton, Ontario, Canada, and Hainan Medical College, Haikou, Hainan, China; aa Department of Laboratory Medicine, Clinical Center, NIH, Bethesda, Maryland, USA; bb Johns Hopkins University School of Medicine, Baltimore, Maryland, USA; University of Texas Health Science Center

**Keywords:** Cryptococcosis, *Cryptococcus gattii*, *Cryptococcus neoformans*, clade, genetic diversity, new nomenclature, species complex

## Abstract

Cryptococcosis is a potentially lethal disease of humans/animals caused by *Cryptococcus neoformans* and *Cryptococcus gattii*. Distinction between the two species is based on phenotypic and genotypic characteristics. Recently, it was proposed that *C. neoformans* be divided into two species and *C. gattii* into five species based on a phylogenetic analysis of 115 isolates. While this proposal adds to the knowledge about the genetic diversity and population structure of cryptococcosis agents, the published genotypes of 2,606 strains have already revealed more genetic diversity than is encompassed by seven species. Naming every clade as a separate species at this juncture will lead to continuing nomenclatural instability. In the absence of biological differences between clades and no consensus about how DNA sequence alone can delineate a species, we recommend using “*Cryptococcus neoformans* species complex” and “*C. gattii* species complex” as a practical intermediate step, rather than creating more species. This strategy recognizes genetic diversity without creating confusion.

## INTRODUCTION

Cryptococcosis is one of the most serious fungal diseases encountered by immunocompromised patients, particularly those with AIDS, throughout the world. The disease is caused by two pathogenic members of the genus *Cryptococcus*, *C. neoformans* and *C. gattii*, and claims an estimated 625,000 lives annually, with a global burden of nearly 1 million cases per year (1, 2). Initially, the two etiologic agents were classified as one species but were distinguished by their antigenic diversity; *C. neoformans* strains are of serotypes A and D, and *C. gattii* strains are of serotypes B and C (3–6). The discovery of two different teleomorphs, one for *C. neoformans* and the other for *C. gattii* (5, 6), ultimately led to the recognition of two species, which was later verified by whole-genome sequence data (7).

As the phylogenetic species concept became widely accepted from the late 1990s, phylogenetic trees constructed on the basis of multilocus sequence typing (MLST) and other molecular typing techniques, such as amplified fragment length polymorphism (AFLP) analysis, showed that both *C. neoformans* and *C. gattii* strains were composed of multiple genetically diverse monophyletic clades totaling 7 to 9 (8–14). Recently, a proposal was made to designate seven MLST clades identified among 115 strains of *C. neoformans* and *C. gattii* into new species: *C. neoformans* into two species and *C. gattii* into five species (14). We believe this proposal to be premature for the following reasons, to be further expanded upon below. (i) Phylogenetic species designation will almost certainly change, since a sample of less than 5% of the genotyped strains poorly represents the true diversity within the species complex. (ii) The use of lineage alone to designate species without readily identifiable phenotypic characteristics that distinguish the species is highly controversial and raises an unsettled issue of how different genomes should be used in delineation of a species. (iii) Solely using cladistic (phylogenetic) approaches for species delineation in cryptococcosis agents is inappropriate since they show various rates of recombination, clonality, and hybridization within and among lineages. (iv) Renaming important pathogens requires a consensus within the scientific community to prevent confusion in the published literature as well as to avoid confusion in clinical practice. This consensus has not yet been achieved.

## GENETIC DIVERSITY WITHIN THE TWO SPECIES AND THE RECENT PROPOSAL OF SEVEN SPECIES NAMES

*C. gattii* strains were once considered a monophyletic clade, but phylogenetic studies based on a concordance of genealogies using 6 to 11 unlinked loci have suggested that *C. gattii* strains are a complex of multiple phenotypically cryptic species (8–12, 14, 15), which is typical of an evolving population. This complexity is also displayed by *C. neoformans* (9, 11, 12, 14, 16). The most commonly used MLST scheme includes seven concatenated loci: *CAP59*, *GPD1*, *IGS1*, *LAC1*, *PLB1*, *SOD1*, and *URA5*, which were recommended by the International Society for Human and Animal Mycology (ISHAM) Genotyping Working Group of *Cryptococcus neoformans* and *C. gattii* (17). The total number of monophyletic clades recognized within the *C. neoformans/C. gattii* species complex is increasing as more strains collected globally are being included in phylogenetic analyses (18). The major monophyletic clades for the two species have most commonly been designated molecular types VNI (AFLP1), VNII (AFLP1A/IB), VNIII (AFLP3), and VNIV (AFLP2) for *C. neoformans* and molecular types VGI (AFLP4), VGII (AFLP6), VGIII (AFLP5), and VGIV (AFLP6) for *C. gattii*. The recent proposal for naming 7 separate species, excluding diploid/aneuploid hybrids formed between different clades based on MLST data of 115 isolates, is as follows (Table 1): *C. neoformans* would be divided into *C. neoformans* (serotype A, VNI/AFLP1 and VNII/AFLP1A, AFLP1B,VNB, formerly *C. neoformans* var. *grubii*), *C. deneoformans* (serotype D, VNIV/AFLP2, formerly *C. neoformans* var. *neoformans*), and a *C. neoformans* × *C. deneoformans* hybrid (formerly VNIII/AFLP3 or AD hybrids). *C. gattii* would be recognized as five separate species, namely, *C. gattii* (VGI/AFLP4), *C. deuterogattii* (VGII/AFLP6), *C. bacillisporus* (VGIII/AFLP5), *C. tetragattii* (VGIV/AFLP7), and *C. decagattii* (VGIV and VGIIIc/AFLP10). The diploid/aneuploid hybrids between isolates of the *C. neoformans* and *C. gattii* complexes are named a *C. deneoformans* × *C. gattii* hybrid (AFLP8), a *C. neoformans* × *C. gattii* hybrid (AFLP9), and a *C. neoformans* × *C. deuterogattii* hybrid (AFLP11). A diligent search by us failed to find a correlation between the new species name and phenotypic characteristics. Susceptibility to antifungal agents, biochemical markers, virulence based on experimental animals, or prevalence in patients with distinct underlying conditions revealed some tendencies but were sufficiently varied to be unreliable for differentiation among species. Matrix-assisted laser desorption ionization–time of flight (MALDI-TOF) mass spectrometry was used on 423 isolates which had been molecularly divided into the proposed species. Using an in-house database, almost all isolates were identified to the correct species during at least one of two duplicate trials using a 1.7 cutoff. However, this low cutoff is usually used for genus and not for species recognition (19). Only 76.1% of strains were identified correctly by both tests using the usual species cutoff of 2.0, which questions the practicality of this method of species identification (14). The pros and cons of adopting the new species system are noted below.

**TABLE 1:** Recently proposed new names for *C. neoformans* and *C. gattii* species complexes^a^

Current name	Molecular type(s)	Proposed name
*C. neoformans*		
Var. *grubii*	VNI/VNII/VNB (AFLP1, AFLP1A, AFLP1B,VNB)	*C. neoformans*
Var. *neoformans*	VNIV (AFLP2)	*C. deneoformans*
AD hybrid	VNIII (AFLP3)	*C. neoformans* × *C. deneoformans* hybrid

*C. gattii*	VGI (AFLP4)	*C. gattii*
	VGII (AFLP6)	*C. deuterogattii*
	VGIII (AFLP5)	*C. bacillisporus*
	VGIV (AFLP7)	*C. tetragattii*
	VGIV/VGIIIc (AFLP10)	*C. decagattii*

DB hybrid	AFLP8	*C. deneoformans* × *C. gattii* hybrid
AB hybrid	AFLP9	*C. neoformans* × *C. gattii* hybrid
AB hybrid	AFLP11	*C. neoformans* × *C. deuterogattii* hybrid

a See reference 14.

## BENEFITS OF ADOPTING THE NEW SPECIES SYSTEM

Because the taxonomic rank of species occupies a pivotal position in every aspect of biology, adoption of a cryptococcal species recognition system that is compatible with the advances in phylogenetic theory is critical. The proposed seven species designations (excluding the four hybrid clades), if generally accepted, would be an important step in formally recognizing the complex biodiversity within the etiologic agents of cryptococcosis. Since clinically relevant biological differences between genetically diverged cryptic species are not always obvious, assigning species names to each clade might accelerate discovery of genetically defined phenotypic differences.

## DISADVANTAGES IN ADOPTING THE NEW CLASSIFICATION SYSTEM AT THIS JUNCTURE

### (i) An insufficient number of isolates was studied.

One of the most important concerns is that the proposed species delineation for the etiologic agents of cryptococcosis has resulted from an MLST-based phylogenetic analysis of 115 strains (<5% of MLST-genotyped strains). Furthermore, one of the new species, *C. decagattii*, was described based on only two strains that were identical by MLST (14) and that may have originated from the same patient. Differing algorithms with larger numbers of isolates may divide clades differently. A recent analysis, which included 2,606 strains, already showed more genetic diversity than is encompassed by seven species (20). A strict, accepted phylogenetic species concept defines a species as a single lineage of ancestor-descendant populations which maintains its identity from other such lineages and which has its own evolutionary tendencies and historic fate (21–23). With this definition, even the smallest diagnosable cluster of individual strains that form a monophyletic group in a phylogenetic tree can be considered deserving of species recognition (24), and the number of cryptococcosis agents with a species status will continue to increase (18). For this reason, the proposed taxonomy is likely to prove to be unstable.

### (ii) More of the genome needs to be represented.

Since the 11 loci used for the MLST-based phylogenetic tree represent only 43% of the cryptococcal chromosomes (6 of 14 chromosomes) (14), the true extent of diversity and recombination events will not be known until more of the chromosomes are included. For example, whole-genome sequencing recently identified hitherto-unmapped levels of genomic diversity and population genetic structure among clinical and environmental isolates of *C. neoformans* in Africa (25) and led to the discovery of new lineages. Further, until whole-genome sequencing was carried out, gene introgression from *C. neoformans* var. *grubii* (VNI) to the Pacific Northwest population of *C. gattii* (old name) strains was not recognized (26). This observation was also the case with gene introgression from *C. neoformans* var. *grubii* to *C. neoformans* var. *neoformans* (27). Although these findings of gene introgression do not change the broad-scale phylogenetic relationships, the findings illustrate our poor understanding of genetic exchange between different clades. We need further genome-wide studies to uncover this basic information about recombination for delimiting species boundaries.

### (iii) Models applied for species delineation may not be appropriate.

Delineation of seven species (Table 1) was based on models derived for sexually reproducing and freely recombining organisms, such as birds, bats, and certain insects (14). As *C. neoformans* and *C. gattii* more typically reproduce clonally, the algorithms used may not be appropriate and may tend to be biased toward declaring clonal lineages as species.

### (iv) Species designations are too complex (i.e., routine identification is impractical).

Sequencing 11 loci and constructing a phylogenetic tree would need to be replaced by simpler techniques for routine use, even in reference laboratories. MALDI-TOF mass spectrometry was too imprecise, particularly for hybrid species (14). A universally used molecular method of fungal species identification is determination of the nuclear ribosomal internal transcribed spacer (ITS) sequence (28, 29). However, this option is not available for the identification of seven species due to insufficient ITS sequence variation among the clades/species within either *C. neoformans* (old name) or *C. gattii* (old name) (14, 30).

### (v) Species names are confusing.

Significant confusion will result from using the names “*gattii*” and “*neoformans*” in two different contexts. Until 2015, the name *C. gattii* was used for all the strains of serotypes B and C belonging to the VGI to VGIV molecular types. The same name in the new system refers only to those belonging to the VGI molecular type (14). This change will cause a disconnection between new *C. gattii* strains and prior clinical information on diagnosis, the progression of disease, and underlying risk factors of the patients infected with old *C. gattii* strains. This disconnection is of particular concern because considerable work on clinical strains and basic research on *C. gattii* was carried out using the Vancouver epidemic reference strain R265 (VGII), which is now proposed as a strain of *C. deuterogattii* (14). Strain R265 is highly virulent but is not as neurotropic as other strains of *Cryptococcus* (31, 32), and many features of *C. gattii* learned from using strain R265 may not be applicable to the new *C. gattii* strains. In addition, since new names break apart the former *C. gattii* strains, the common properties shared by the cryptic species will be lost. The word “*neoformans*” has been used for 2 decades to identify not only a species but also a variety (*C. neoformans* var. *neoformans*). The name “*C. neoformans*” in the new system refers only to strains of serotype A and molecular types VNI and VNII/VNB and will cause considerable confusion in referencing the existing results.

### (vi) Names for hybrid and aneuploid strains are not readily accommodated.

There are diploid or aneuploid hybrid strains formed by fusion of the strains into two different clades, such as serotype AD (VNI/VNIV) hybrids (33, 34) and serotype AB (VNI/VGI) hybrids (35). The frequency of *C. neoformans* AD hybrid strains among global clinical isolates is reported to be 6%, slightly higher than that of the VNIV molecular type (5%) (11), and it is considerably higher (30%) among European clinical isolates (13). The new name, “*C. neoformans* × *C*. *deneoformans* species hybrid” instead of “AD hybrids” or “VNIII” will be impractical to use for the strains with such frequency. Furthermore, the identification of AD hybrids by MALDI-TOF mass spectrometry has been inconsistent (14), and we also do not know whether the MALDI-TOF protein profiles of AD aneuploid/diploid hybrids are distinguishable from the homoploid hybrids (34, 36, 37) formed by mating between VNI (new name, *C. neoformans*) and VNIV (new name, *C. deneoformans*) strains. Though recombinant haploids are infrequent, recent MLST studies have identified putative recombinant haploids formed between VNI strains and VNIV strains among clinical and environmental isolates (38). The homoploid hybrids formed by mating between serotype A and D strains could not be named in the new species system. Finally, aneuploid hybrids may have extensive phenotypic variation, depending on which chromosomes are present in duplicate.

## PROPOSED USE OF “*C. NEOFORMANS* SPECIES COMPLEX” AND “*C. GATTII* SPECIES COMPLEX”

“Species complex” in biology usually implies that two or more cryptic species are hidden under one species name, which makes both *Cryptococcus neoformans* and *C. gattii* typical species complexes. Unlike a “species,” a “complex” has no nomenclatural status and requires no name change. However, the species complexes are clearly defined by conventional diagnostic methods that can be validated by molecular data. The term “species complex” has also served the nomenclatural stability of other fungal taxa, including *Fusarium* species complex (39) and *Scedosporium* species complex (40).

## CONCLUSIONS

Considering the high global burden of this potentially fatal infection, names given to the etiologic agents causing cryptococcosis are of paramount importance for both the mycological community and the medical community. The proposal to divide the two cryptococcosis agents into 7 haploid and 4 aneuploid/diploid hybrid species deserves extensive discussion prior to adoption. Since the seven new species are not known to be clinically distinguishable, universal adoption of the new system of nomenclature should be delayed until more-detailed studies employing a larger number of isolates reveal the clinical and biological relevance of the new species. Adoption of the proposed nomenclature at this juncture might separate taxonomy from clinical practice and in doing so inhibit the progress of both fields. Instead of “species,” “species complex” would accommodate already-known cryptic species and those that might be discovered in the future. Molecular types within each species complex can be designated by their molecular type (VNI/AFLP1, VGI/AFLP4, etc.) whenever necessary. Once clinical and biological relevance becomes apparent for new species distinctions, both mycologists and clinicians will benefit by using new names.
